# Protective practices against zoonotic infections among rural and slum communities from South Central Chile

**DOI:** 10.1186/s12889-015-1964-2

**Published:** 2015-07-28

**Authors:** Meghan R. Mason, Marcelo Gonzalez, James S. Hodges, Claudia Muñoz-Zanzi

**Affiliations:** Division of Epidemiology and Community Health, School of Public Health, University of Minnesota, 1300 S. Second Street, 55454 Minneapolis, MN USA; Instituto de Patologia Animal, Universidad Austral de Chile, Valdivia, Chile; Division of Biostatistics, School of Public Health, University of Minnesota, Minneapolis, MN USA

**Keywords:** Zoonoses, Prevention, Education programs, Social ecology, Community type, Chile

## Abstract

**Background:**

Despite well-recognized recommendations to reduce human exposure to zoonotic pathogens, the use of personal and herd-level protective practices is inconsistent in communities where human interactions with animals are common. This study assessed household-level participation in rodent- (extermination, proper food storage, trash disposal), occupational- (preventive veterinary care, boot-wearing, glove-wearing), and garden-associated (restricting animal access, boot-wearing, glove-wearing) protective practices in farms, villages, and slums in the Los Rios region, Chile, where zoonotic pathogens are endemic.

**Methods:**

Questionnaires administered at 422 households across 12 communities recorded household-level socio-demographic characteristics and participation in nine protective practices. Household inclusion in the analysis of occupational practices required having livestock and a household member with occupational exposure to livestock (n = 127), and inclusion in analysis of garden practices required having a garden and at least one animal (n = 233). The proportion of households participating in each protective practice was compared across community types through chi-square analyses. Mixed effects logistic regression assessed household-level associations between socio-demographic characteristics and participation in each protective practice.

**Results:**

Most households (95.3 %) reported participation in rodent control, and a positive association between the number of rodent signs in a household and rodent extermination was observed (OR: 1.75, 95 % CI: 1.41, 2.16). Occupational protective practices were reported in 61.8 % of eligible households; household size (OR: 1.63, 95 % CI: 1.17, 5.84) and having children (OR: 0.22, 95 % CI: 0.06, 0.78) were associated with preventive veterinary care. Among eligible households, 73.8 % engaged in protective practices when gardening, and species diversity was positively associated with wearing boots (OR: 1.27, 95 % CI: 1.03, 1.56). Household-level participation in all three protective practices within any exposure category was limited (<10.4 %) and participation in any individual protective practice varied considerably within and across community types.

**Conclusions:**

The levels of participation in protective practices reported in this study are consistent with descriptions in the literature of imperfect use of methods that reduce human exposure to zoonotic pathogens. The wide differences across communities in the proportion of households participating in protective practices against human exposure to zoonotic pathogens, suggests that future research should identify community-level characteristics that influence household participation in such practices.

**Electronic supplementary material:**

The online version of this article (doi:10.1186/s12889-015-1964-2) contains supplementary material, which is available to authorized users.

## Background

Of emerging infectious pathogens, 75 % are considered to be zoonotic – those transmitted between animals and humans [1]. Emerging diseases are of concern for human health because they are either completely novel organisms or are more virulent forms of established pathogens. In both cases, humans initially lack immunity to infection, and provide a continuous source of susceptible hosts for the pathogens [2, 3]. Although not all zoonotic pathogens are emerging infectious diseases, many have wildlife reservoirs, and the increasingly indistinguishable boundary between human civilization and wild animal habitats has resulted in the migration of these pathogens to domestic animals where they have become well-established [3, 4]. Human infection with zoonotic pathogens is therefore both associated with travel to exotic locations and direct contact with wild animal species, as well as mundane animal interactions with household pets, livestock, rodents and environments contaminated with zoonotic pathogens [5, 6].

In addition to the human morbidity and mortality that result from zoonotic infections, the cycle of poverty associated with zoonotic disease transmission is well-documented [7, 8]. High incidence of human and animal cases of zoonotic infections is found in communities that depend on their livestock for food and income [7]. Often, animals are kept close to the living spaces of their owners, contributing to increased contact between animals and humans, as well as contamination in the peri-domestic environment [8–10]. If animals get sick, limited resources exist for veterinary care or care of household members who may subsequently become ill, further straining household finances and perpetuating the poverty cycle [11–15].

One challenge for reducing the human public health disease burden is the variety of direct and indirect transmission routes of zoonotic pathogens from animals to humans [16]. Direct transmission of zoonotic pathogens to humans occurs through contact with body fluids and tissues from sick animals (e.g., rabies and brucellosis), inhalation of aerosolized pathogens excreted in feces, tissues, or body fluids from infected animals (e.g., influenza viruses and *Mycobacterium bovis*), and ingestion of contaminated animal products (e.g., *Salmonella* and *E. coli*) [1, 17]. Indirect transmission results from contact with an environment contaminated with zoonotic pathogens. The persistence of many organisms in the soil, water, or animal excrement, outside of the animal host, allows for frequent human contact opportunities, creating an added layer of uncertainty when trying to identify the infection source. In the case of leptospirosis, arenaviruses, and hantaviruses, the pathogens are passed from the urine and/or feces of rodents and can survive in proper conditions for weeks to months [18, 19].

The complexity and array of zoonotic disease transmission pathways therefore require a comprehensive set of protective practices, in conjunction with the standard prevention measure of proper hand hygiene, to reduce the risk of human infection [20, 21]. Protective practices often promoted to reduce human exposure to zoonotic pathogens include wearing personal protective equipment when working around livestock and in the garden (e.g., gloves and boots) [16, 20, 22, 23], as well as rodent control measures such as proper food handling and storage, trash and waste disposal [24–26]. Within the animal population, the overall burden of zoonotic pathogens can be reduced by a combination of individual and herd-level practices. Vaccination of pets and livestock, treating parasitic infections, quarantining sick animals, and maintaining clean food and water supplies, keep individual animals free from infections [2]. Population-based practices such as routine disease surveillance, participation in eradication efforts, and the maintenance of overall herd health, have been widely recommended to lessen the transmission of zoonotic pathogens among animal populations, which subsequently reduce the risk of transmission to humans [27]. For small and subsistence farmers, control over the movement of animals in the peri-domestic environment is also important to reduce contamination of garden and living spaces with zoonotic pathogens excreted in feces, urine, and other body fluids and tissues of infected animals [27, 28]. The accuracy with which these individual and herd-level protective practices are performed, and their duration and sustainability, vary substantially, contributing to challenges in measuring participation in these practices and evaluating their effectiveness [29–31]. Despite the limitations of these interventions, they are recommended globally as mechanisms to reduce zoonotic disease transmission among animals, and from animals to humans [26].

While the literature is rich in documentation of particular circumstances in which recommended protective practices are performed at the individual and household level to reduce human exposure to zoonotic pathogens, these studies tend to be restricted to disease-specific interventions [32, 33], high-risk occupational settings [20, 34, 35], and responses to outbreaks of zoonoses in flocks and herds [29, 36, 37]. To-date, little attention has been given to describing the routine protective practices carried out by households. Understanding these behaviors is important to inform effective public health programs.

The Los Rios Region in South Central Chile, where this study was conducted, is a temperate climate area in which agriculture and animal husbandry are primary economic interests, involving of a mixture of large and small scale farms. This setting lends itself well to persistent endemic rates in humans of several zoonotic pathogens. For example, the annual incidence rates of trichinosis and echinococcosis are 5.50 and 7.34 per 100,000 persons, respectively [38, 39]. Hantaviruses have an annual incidence rate of 1.31 per 100,000 persons in addition to documented sporadic cases and outbreaks [40], and an estimated seroprevalence of 1.07 % [41]. Toxoplasmosis also is particularly common in the region with 40 % of the human population showing serological evidence of prior infection, of which, a large proportion is attributed to the ingestion of oocysts from the environment [42].

Within this context, this study sought to assess the level of household-level participation in a specific list of protective practices against human infection with zoonotic pathogens. In order to contribute to the understanding of prevention of zoonotic disease transmission in areas of endemic human rates of infection, this study had two specific objectives. The first was to compare household-level participation in rodent- (extermination, proper food storage, trash disposal), occupational- (preventive veterinary care, boot-wearing, glove-wearing), and garden-associated preventive practices (restricting animal access, boot-wearing, glove-wearing) within and across three distinct community types (farm areas, rural villages, and urban slums). The second objective was to assess whether any household socio-demographic characteristics were associated with each of the nine preventive practices.

## Methods

### Selection of study site, community types, and households

The data used for this study were obtained from a broader research project conducted between November 2010 and April 2012 in the Los Rios Region of South Central Chile on the eco-epidemiology of leptospirosis [43, 44]. Surveys were administered by study staff during the spring (September-December) and summer months (January–April) each year. Four communities were selected from each of three community types: marginalized or slum urban communities (n = 142 households), farming areas (n = 146 households), and rural villages (n = 134 households), yielding 422 total households. Marginalized urban communities (referred to as U-1 through U-4) were characterized as informal settlements commonly outside of major cities, where housing conditions were predominantly substandard and the population was highly concentrated. Farm areas (referred to as D-1 through D-4) were classified based on a predominance of dispersed households, mainly small family farms, located in a rural locality. Rural villages (referred to as C-1 through C-4) were considered to be located away from major population centers but with closely spaced households. The twelve communities were selected based on their representativeness of the three types of settings, the preliminary interest of community leaders in participating in the larger study, and their proximity to the local collaborating university. Individual households were selected randomly and enrolled based on willingness to participate.

A particular set of households was used for the analysis of protective practices in each of the three sources of exposure (rodents, occupational settings, and the garden). All 422 households were considered eligible for participation in rodent control practices since all households were at-risk for rodent presence. The analyses of occupation-associated protective practices included only farm households that owned livestock (at least one animal) and had at least one family member with reported contact with livestock (e.g., during the birthing process, when milking or butchering an animal, or when cleaning animal barns) (n = 127). Households which both owned at least one animal (livestock or pet) and had a vegetable garden were considered for assessment of gardening-related protective practices (n = 233) (Table 1).

**Table 1:** Definitions of protective practices asked in household questionnaires and corresponding household inclusion criteria for analyses

Exposure category	Household inclusion criteria (n)	Protective practice	Definition
Rodents	All households (n = 422)	Extermination	Use of traps or poison to eliminate rodents
		Food Storage	Use of a container with a sealed lid for food storage
		Trash Disposal	Use of a covered container for a household trash receptacle such as a bin or bucket with a lid
Occupation	Household owns at least one animal classified as livestock, and at least one household member reported regular contact with livestock (e.g., during the birthing process, milking or butchering animals, or cleaning animal barns) (n = 127)	Preventive Veterinary Care	Use of vaccinations or anti-parasitic treatment for at least one of the animals that the household owns at least once
		Wearing Boots	Wearing rubber boots when working with livestock
		Wearing Gloves	Wearing gloves when working with livestock
Garden	Household has at least one livestock, pet, or other animal and has a vegetable garden (n = 233)	Restricting Animal Access	Preventing livestock and domestic animals from entering the vegetable garden and surrounding area.
		Wearing Boots	Wearing rubber boots when working in the garden
		Wearing Gloves	Wearing gloves when working in the garden

### Household participation in protective practices

At each household, a questionnaire was administered by study staff to collect data on participation in protective practices within the categories of rodent control, occupational protection, and protection during gardening activities. Inclusion criteria for each of the three exposure categories and definitions for each of the corresponding protective practices are listed in Table 1. All nine activities in the three protective practice categories were considered as binary variables; either a household reported participation in a protective practice or they did not.

### Household socio-demographic characteristics

Data collected through the household questionnaire for evaluating associations between socio-demographic factors and participation in protective practices included the sex (male or female), age (in years) and high school graduation status (yes or no) of the head of household. Other factors included the number of people in the household (range 1–12), whether the household had children under age 18 (yes or no), household monthly income (above or below $350 USD, the study population’s median income), and number of animals of each species (pets: dogs, cats, livestock: horses, sheep/goats, cows, pigs, and other: poultry). Species diversity was defined as the total number of different animal species present (range 0–7). A proxy for rodent presence was defined as the number of rodent signs a household reported from the following indicators: seeing rodents, seeing rodent droppings, seeing or smelling rodent urine, seeing gnawed boxes, food, or wood, holes in the walls, or hearing rodent noises (Table 2).

**Table 2:** Socio-demographic characteristics of participating farm, village, and slum households from the Los Rios region, Chile (2010–2012)

	All households (n = 422)	Small village households (n = 134)	Farm households (n = 146)	Marginalized urban households (n = 142)	*P*-value
	n (%)	n (%)	n (%)	n (%)	
Income level^a^					
≤ $350 USD per month	263 (62.3)	78 (58.2)	74 (50.7)	111 (78.2)	<0.01
>$350 USD per month	159 (37.7)	56 (41.8)	72 (49.3)	31 (21.8)	
Head of household education^a^					
< High school	311 (73.7)	95 (70.9)	106 (72.6)	110 (77.5)	0.43
High school or greater	111 (26.3)	39 (29.1)	40 (27.4)	32 (22.5)	
Sex head of household^a^					
Male	159 (37.7)	54 (40.3)	76 (52.1)	29 (20.4)	<0.01
Female	263 (62.3)	80 (59.7)	70 (47.9)	113 (79.6)	
Age head of household^a^					
Mean (range)	45.6 (17–87)	48.6 (19–85)	51.5 (18–87)	36.7 (17–77)	<0.01
Any children in the household^a^					
Yes	285 (67.5)	78 (58.2)	95 (65.0)	112 (78.9)	<0.01
No	137 (32.5)	56 (41.8)	51 (35.0)	30 (21.1)	
Number of people in the household^a^					
Mean (range)	4.2 (1–12)	4.1 (1–12)	4.4 (2–12)	4.1 (1–11)	0.18
Species diversity^a^					
Mean (range)	2.7 (0–7)	2.7 (0–7)	4.0 (0–7)	1.3 (0–3)	<0.01
Household owns dogs					
Yes	345 (81.8)	113 (84.3)	134 (91.8)	98 (69.0)	<0.01
No	77 (18.2)	21 (15.7)	12 (8.2)	44 (31.0)	
Household owns cats					
Yes	235 (55.7)	77 (57.5)	87 (59.6)	71 (50.0)	0.23
No	187 (44.3)	57 (42.5)	59 (40.4)	71 (50.0)	
Household owns livestock					
Yes	195 (46.2)	61 (45.5)	131 (89.7)	3 (2.1)	<0.01
No	262 (53.8)	73 (54.5)	15 (10.3)	139 (97.9)	
Household has a garden					
Yes	242 (57.3)	91 (67.9)	122 (83.6)	29 (20.4)	<0.01
No	180 (42.7)	43 (32.1)	24 (16.4)	113 (79.6)	
Type of house					
Shack/hut	123 (29.1)	3 (2.2)	2 (1.4)	118 (83.1)	<0.01
House	299 (70.9)	131 (97.8)	144 (98.6)	24 (16.9)	
Condition of house					
Good	349 (82.7)	116 (86.6)	132 (90.4)	101 (71.1)	<0.01
Floors, walls, and/or roof deteriorated	73 (17.3)	18 (13.4)	14 (9.6)	41 (28.9)	
Drinking water source					
Community or household tap	311 (73.7)	121 (90.3)	49 (33.6)	141 (99.3)	<0.01
Well	68 (16.1)	3 (2.2)	65 (44.5)	0	
Natural water source	43 (10.2)	10 (7.5)	32 (21.9)	1 (0.7)	
Human waste disposal					
Septic tank/system	232 (55.0)	108 (80.6)	85 (58.2)	39 (27.5)	<0.01
Latrine	110 (26.0)	21 (15.7)	50 (34.2)	39 (27.5)	
None/Outhouse	80 (19.0)	5 (3.7)	11 (7.5)	64 (45.0)	
Garbage removed by truck					
Yes	363 (86.0)	119 (88.8)	109 (74.7)	135 (95.1)	<0.01
No	59 (14.0)	15 (11.2)	37 (27.2)	7 (4.9)	
Number of rodent signs^a^					
Mean (range)	1.7 (0–5)	1.6 (0–4)	2.0 (0–5)	1.5 (0–5)	<0.01
Survey season					
Summer (January–April)	161 (38.2)	63 (47.0)	65 (44.5)	33 (23.2)	<0.01
Spring (September–December)	261 (61.8)	71 (53.0)	81 (55.5)	109 (76.8)	

^**a**^Used as independent variables in regression analyses for participation in protective practices against zoonotic disease transmission

Additional infrastructure characteristics of the household collected by the questionnaire but not used for the analyses of socio-demographic characteristics and participation in protective practices are listed in Table 2. Surveys were administered during the spring (September–December) and summer months (January–April) each year.

The study protocol was approved by the University of Minnesota’s Institutional Review Board (No. 0903 M62042), the Institutional Animal Care and Use Committee (No. 0904A63201), and Austral University’s Human and Animal Ethics Committee (No. 01/09).

### Data analyses

After examining the distribution of socio-demographic characteristics across community types, a separate but identical set of analyses was performed for each of the three categories of protective practices (rodent control, occupational protection, and protection in the garden). The proportion of households engaging in each of the practices was examined overall, by community, and across community types. Differences across community types in the proportion of households participating in protective practices were assessed using Chi-square tests or Fisher’s tests as appropriate [45].

Logistic regression models were developed to examine associations between household socio-demographic factors, and participation in each of the nine zoonotic protective practices. Both univariate and multivariate mixed models were constructed with random intercepts included at the community level to take into consideration potential correlations of responses within communities. Stepwise model selection was used, along with examination of the Akaike information criterion (AIC) to construct a multivariate model for each protective practice. Confounding and effect modification were then considered for the variable of community type, and other socio-demographic factors originally omitted from the multivariable model but considered to be potential mechanisms necessary to understanding the factors associated with household participation in the protective practices. Odds ratios (OR) and 95 % confidence intervals (CI) for each statistically significant (α = 0.05) socio-demographic factor included in the final model were calculated following standard methods [46]. Regression analyses were performed in R version 2.15 using the lme4 package [47, 48].

## Results

### Study population

The distribution of the socio-demographic characteristics reported in the household questionnaire varied substantially by community type (Table 2). The farm and rural village households had higher incomes, older heads of households; more often had children, and had greater species diversity than marginalized urban households. Marginalized urban households were less likely to have gardens, have a male head of household, live in permanent houses, or have good household infrastructure than the farm or small village communities. While the marginalized urban households were least likely to have a septic tank or system for human waste disposal, they were most likely to have their garbage picked up by trucks, and had the fewest rodent signs per household on average.

### Participation in rodent control practices

#### Comparisons of participation in rodent control practices by community type

**Fig. 1:**
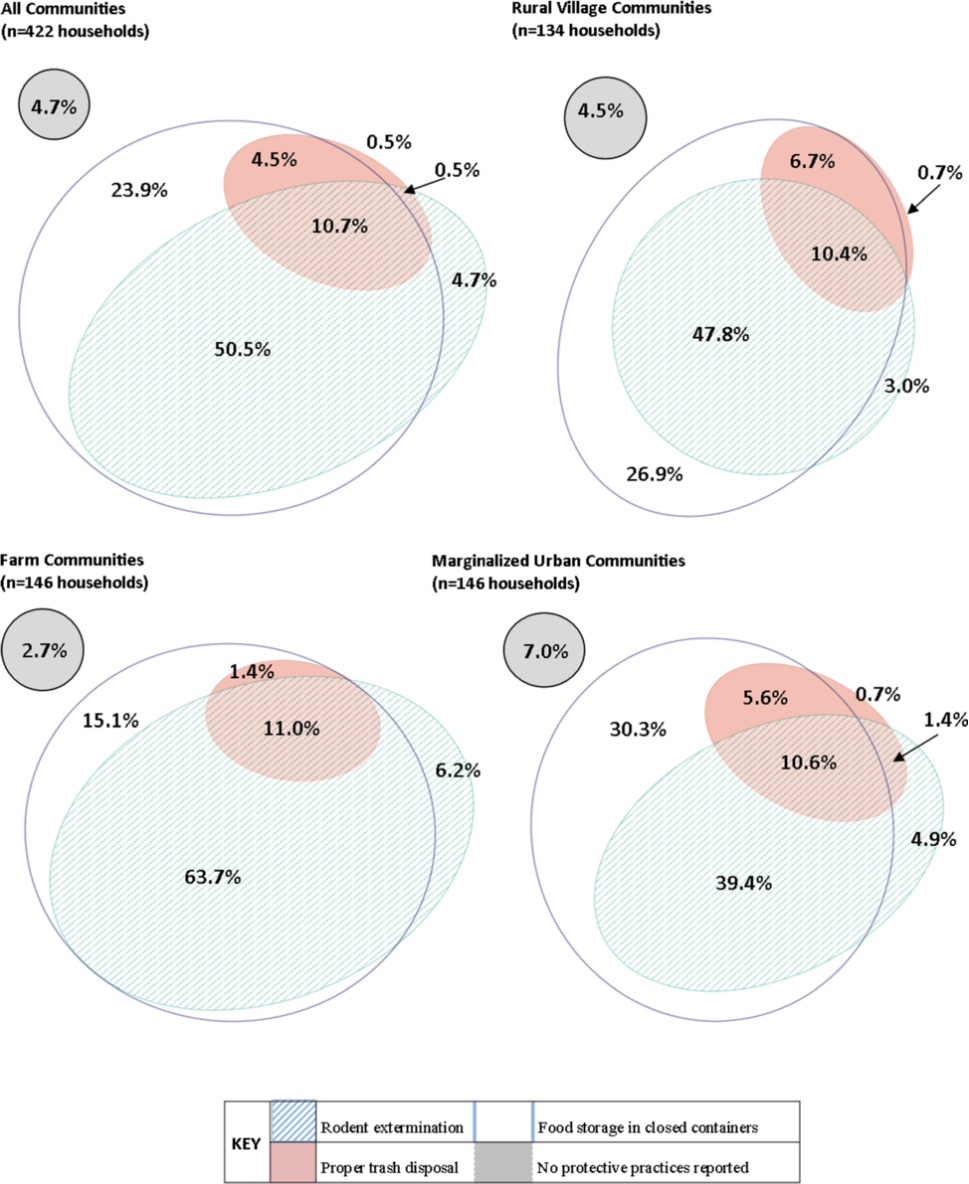
Households participating in rodent control practices, Los Rios region, Chile (2010–2012). The proportion of households reporting participation in rodent control protective practices: extermination, proper food storage, and trash disposal is shown overall, and by community type

Overall, 95.3 % (402/422) of households reported participating in at least one rodent control practice. The most common rodent control activity was storing food in closed containers (89.6 %, 378/422), followed by rodent extermination (66.4 %, 280/422), and proper trash disposal (16.1 %, 68/422) (Fig. 1). Based on chi-square tests, the three community types did not differ significantly in the proportion of households keeping food in closed containers (*P* = 0.31) or practicing proper trash disposal (*P* = 0.37). However, significantly more households in the farm communities participated in rodent extermination compared to rural villages and marginalized urban communities (*P* < 0.01). This difference was also observed at the community level where communities D-2 and D-4 had the highest proportion of households participating in rodent extermination (90.1 % and 85.7 %, respectively) and U-4 had the lowest (37.5 %) of all twelve communities. The contrast between individual farm communities and rural villages was less pronounced (Additional file 1).

Participation in all three rodent control practices was consistent across all three community types with 11.0 % of farms, 10.4 % of rural village households, and 10.6 % of marginalized urban households reporting participation in all three protective practices (Chi-square *P* = 0.99). In farm communities, only 2.7 % of households reported that they did not participate in any protective practice compared to 7.0 % of marginalized urban households and 4.5 % of rural village households, but this difference was not statistically significant (Chi-square *P* = 0.23) (Fig. 1).

Use of cats for rodent control was not included as a protective practice as it is not endorsed as an effective method for reducing zoonotic disease transmission, but cat ownership was included in the household survey. Of the 235 households with cats, 171 (72.8 %) reported that they used cats as a method of rodent control, and 85 (36.2 %) reported it as their only method of rodent control. The proportion of households participating in rodent extermination was lower in households with cats (61.2 %) than in households without cats (72.7 %) (Chi-square *P* = 0.02).

#### Associations between socio-demographic factors and rodent extermination

The multivariable analysis for rodent extermination yielded a final model that included number of rodent signs (*P* < 0.01) while adjusting by community type (Table 3). An increase of one rodent sign was associated with a 1.75-fold increase in the odds of participation by the household in rodent extermination (OR: 1.75, 95 % CI: 1.41, 2.16). Among households reporting no rodent signs, participation in rodent extermination was 37.0 %. When one rodent sign was reported in a household, 63.2 % reported participating in rodent extermination, which increased to 70.0 % of households when two rodent signs were reported.

**Table 3:** Results of the mixed-effects logistic regression analyses for associations between socio-demographic characteristics and protective practices. Only practices associated with at least one statistically significant socio-demographic factor are shown

Exposure category	Protective practice	Socio-demographic characteristic	Odds ratio (95 % CI)	P-value
Rodents	Extermination	Number of rodent signs	1.75 (1.41–2.16)	<0.01
		Community type^a^		
		*Farm areas*	2.31 (1.32–4.20)	<0.01
		*Marginalized urban*	0.85 (0.51–1.40)	0.52
	Trash disposal	Number of people in household	1.17 (1.02–1.34)	0.03
Occupation	Preventive veterinary care	Number of people in household	1.65 (1.20–2.27)	<0.01
		Household has children	0.20 (0.06–0.70)	0.01
Garden	Restricting animal access	Age of head of household^b^	1.24 (1.04–1.48)	0.02
		Community type^a^		
		*Farm areas*	1.97 (1.07–3.63)	0.03
		*Marginalized urban*	0.35 (0.11–1.11)	0.07
	Wearing boots	Species diversity	1.27 (1.03–1.56)	0.03

^a^Village was used as reference category

^b^Odds ratio for an increase of 10 years

#### Associations between socio-demographic factors and proper trash disposal and food storage

In the regression analysis, the only significant factor associated with proper trash disposal was the number of people per household (Table 3). The addition of one member to the household increased the odds of participation in proper trash disposal by 17 % (OR: 1.17, 95 % CI: 1.02, 1.34, *P* = 0.03). No confounding or effect modification was observed in the analysis of this protective practice, but variation across the twelve individual communities for participation in this protective practice was non-negligible. A difference of one standard deviation in the random intercept for community increased the odds of participation in proper trash disposal more than increasing the family size by one person. The proportion of households participating in proper trash disposal was highest in community U-3 (40.0 %) and lowest in community U-4 (6.3 %) demonstrating marked variation even within the same community type (Additional file 1).

No statistically significant associations were noted between socio-demographic factors and whether a household stored food in closed containers. This practice was frequent in all communities with between 72.5 % (U-1) and 96.7 % (C-1) of households reporting keeping their food in closed containers (Additional file 1).

### Preventive veterinary care and occupational protective practices

**Fig. 2:**
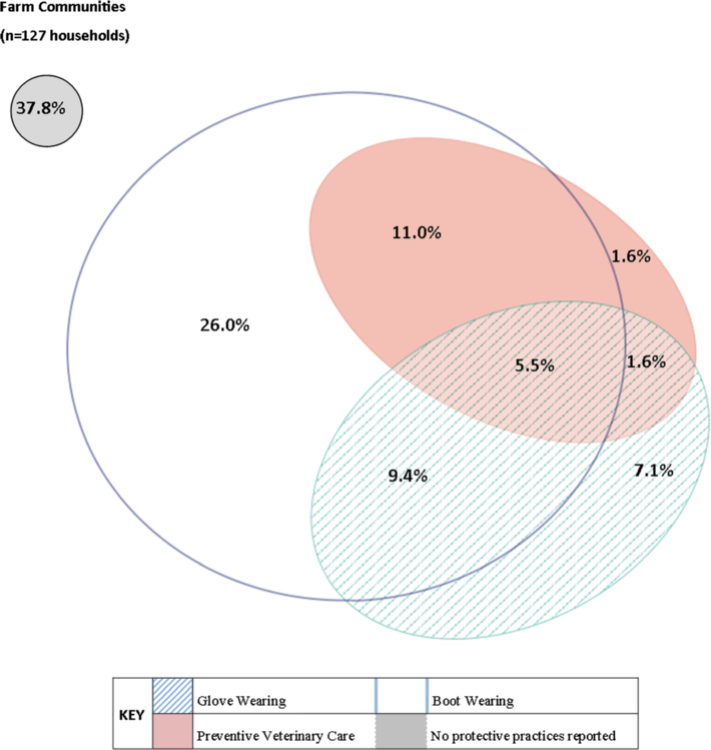
Households participating in occupational protective practices, Los Rios region, Chile (2010–2012). The proportion of households reporting participation in occupational protective practices: preventive veterinary care, wearing gloves, and wearing boots practices is presented for eligible farm households. Eligible households reported owning at least one livestock animal, and at least one family member had regular contact with livestock

More than half of eligible households reported participation in at least one occupational protective practice (62.2 %, 79/127). The most common protective practice was the use of boots (51.9 %), followed by wearing gloves (23.6 %) and preventive veterinary care (19.7 %). Household engagement in joint occupational prevention practices was limited; with only seven households (5.5 %) reporting participation in all three practices (Fig. 2).

#### Associations between socio-demographic factors and participation in occupational protective practices

No socio-demographic factors were significantly associated with the use of boots or gloves. For the outcome of preventive veterinary care, the addition of one family member increased the odds of participation 1.65-fold (95 % CI: 1.20, 2.27) (Table 3). This multivariate model also found that households with children were less likely to participate in preventive veterinary care than those without children (OR: 0.20, 95 % CI: 0.06, 0.70) (Table 3). Although no confounding or effect modification was observed in the analysis of this protective practice, the mixed model did indicate that there were differences across the farm communities. The effect of a one standard deviation increase in the community’s random intercept increased the odds of participation in preventive veterinary care more than the addition of one family member to the household. This was further confirmed by the wide range in the proportion of households reporting participation in preventive veterinary care across the four farm communities (0.0 % to 38.5 %, Additional file 1).

Although species diversity was not a significant factor associated with preventive veterinary care, differences were observed when examining the presence and absence of specific species and whether a household participated in preventive veterinary care. Notably, all of the 25 households that participated in preventive veterinary care owned at least one cow, representing 26.3 % of households with cows. Households with horses had the highest proportion of participation in preventive veterinary care (33.3 %, 7/21) while the lowest proportion was found among households with sheep or goats (13.9 %, 11/79). Households with at least 20 individual livestock animals, regardless of species, were also more likely to participate in preventive veterinary care than those households with fewer than 20 livestock animals (28.9 % v. 14.6 %, respectively), but the difference was not statistically significant (Chi-square *P* = 0.09).

### Participation in protective practices in the garden

#### Comparisons of participation in protective practices in the garden by community type

**Fig. 3:**
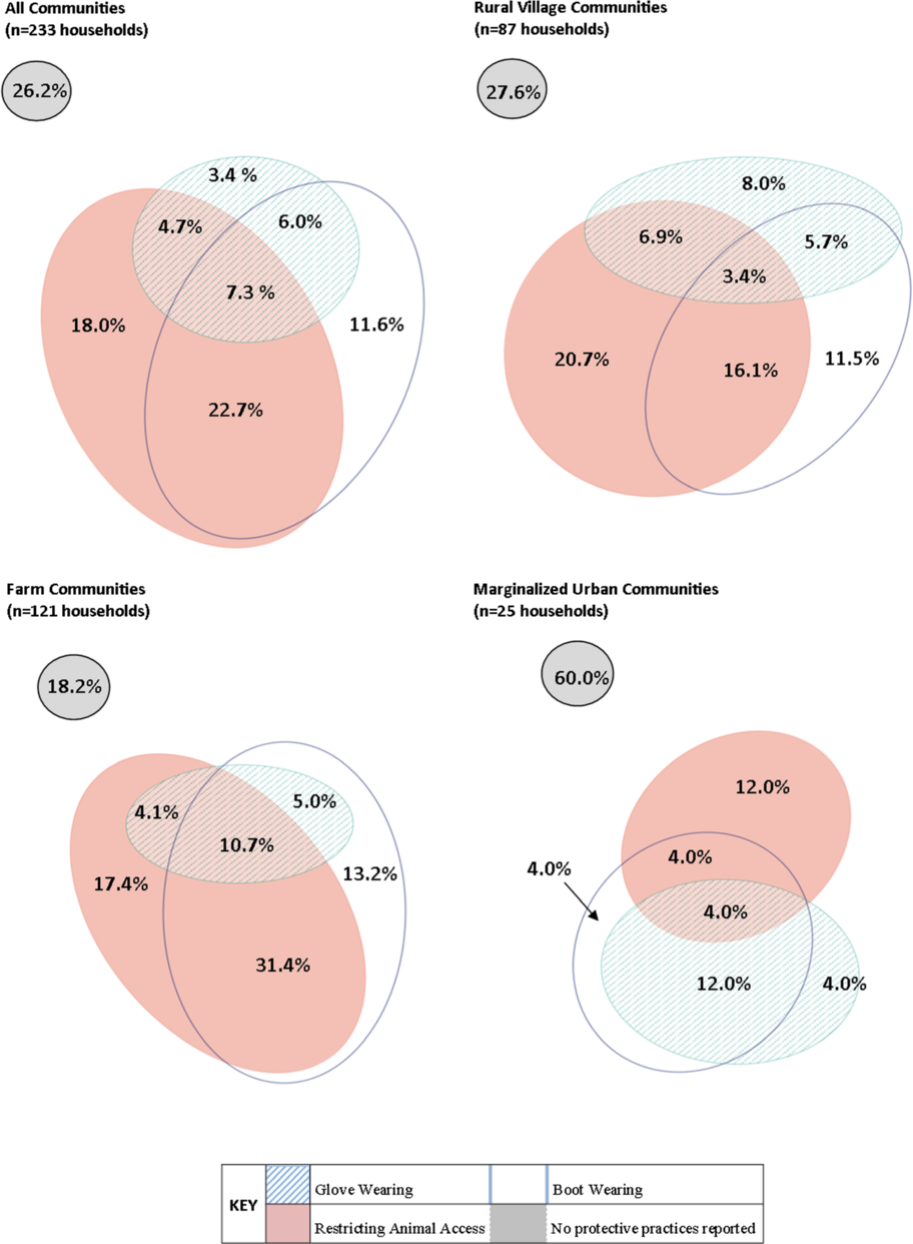
Households participating in protective practices in the garden, Los Rios region, Chile (2010–2012). The proportion of eligible households reporting participation in protective practices in the garden: restricting animal access, wearing gloves, and wearing boots when working in the garden, are presented is presented overall, and by community type. Eligible households reported having a garden and owning at least one livestock, pet, or other animal

Of the households that both owned animals and had a vegetable garden, most were likely to report participation in at least one protective practice in the garden (73.8 %, 172/233). Participation in multiple prevention practices was more likely than participating in only one practice (40.7 % and 33.0 %, respectively), with the most commonly reported combination being wearing boots and restricting animal access to the garden (Fig. 3). The farm community type had the highest proportion of households participating in all three protective practices in the garden (10.7 %), but this did not differ significantly from the proportion of households in rural villages (3.4 %) and marginalized urban communities (4.0 %) (Fig. 3). Urban communities were more likely than rural villages and farm communities to have households that did not participate in any prevention activities in the garden (*P* < 0.01) (Fig. 3). The use of boots in the garden was more commonly reported by farm households compared to other community types (*P* < 0.01) as well as prohibiting animal access to the garden (*P* < 0.01). Community types did not differ significantly in the proportion of households reporting glove use (*P* = 0.74).

#### Associations between socio-demographic factors and participation in protective practices in the garden

Because only 25 urban households had gardens and animals, one random intercept was used to represent households in the urban community type instead of four community-level intercepts in this set of regression analyses. For the outcome of preventing animal access to the garden, the head of household’s age was the only statistically significant factor, and community type was included as a confounder. A ten-year increase in the head of household’s age was associated with a 1.24-fold increase in the odds of preventing animal access to the garden (OR: 1.24, 95 % CI: 1.04, 1.48) (Table 3).

Species diversity was the only independent predictor of a house reporting use of boots when working in the garden (OR: 1.27, 95 % CI: 1.03, 1.56). No confounding or effect modification was observed in the analysis (Table 3). The mixed-effects regression model suggested that the unexplained variance between communities was large when examining wearing boots in the garden. A one standard deviation increase in a community’s random intercept increased the odds of wearing boots while gardening by 3.07, which was larger in magnitude than the odds ratio for the presence of one additional animal species at a given household. This was further confirmed by the spread of the proportion of households reporting the practice in each community, with as few as 0 % (U-2) and as many as 91.2 % (D-1) of households wearing boots in the garden (Additional file 1).

Among households that were included in both the analysis of occupational and garden protective practices (n = 108), boot-wearing in one setting was indicative of boot-wearing in the other setting (Chi-square *P* < 0.01). Of households that reported boot wearing in the garden, 69.7 % also reported boot wearing for occupational protection. Of the households that did not wear boots in the garden, 26.2 % of reported using boots for occupational protection. No socio-demographic variables were associated with use of gloves in the garden.

## Discussion

### Protective practices against human exposure to zoonotic pathogens

This study documented household participation in nine protective practices against zoonotic disease transmission in the Los Rios region, Chile from three common exposure sources: rodents, occupational settings, and the garden. To the best of the authors’ knowledge, no outbreaks of zoonotic pathogens in humans in the study area were present during data collection. The level of participation reported in this study is therefore considered to be representative of routine engagement in protective practices.

Consistent with other studies on protective practices, the proportion of people reporting that they engaged in these globally recommended practices depends on the particular practice. Other studies on personal protective equipment that also measured glove wearing found as few as 0.87 % of households with backyard chickens used gloves as protective measures against avian influenza in Thailand, and a minority of swine and poultry farmers in Minnesota [20, 29]. Glove wearing in this study ranged from 7.1 % to 46.2 % for households with potential occupational exposure to zoonotic pathogens, and from 0.0 % to 80.0 % for households with potential exposure in the garden (Additional file 1). Boot use is more common than the use of gloves as a protective practice. Previous studies have reported that 78 % and 84 % of swine workers in Thailand and Romania, respectively, used boots as a protective practice [34, 49], while this study observed a wide range of participation (3.6 % to 78.4 %). Because the potential for exposure to zoonotic pathogens can be pervasive, it is important that those at-risk use all available recommended measures [6].

Within each category, study results showed that few households reported engaging in all three protective practices simultaneously. This is of concern because zoonotic diseases have many transmission routes, and comprehensive protection is important for reducing human infection risk [12]. For example, rodent control of some type was nearly universal (95.3 %), and most people stored food in closed containers, but by not engaging in proper trash disposal or extermination efforts in conjunction with proper food storage, rodents may still be present in the household. In the case of occupational exposure, many households did not participate in any protective measures (37.8 %), and only 5.5 % of households reported engaging in all three protective practices. The low participation in occupational protection practices is particularly interesting because most of the recommendations for zoonotic disease prevention are written for those who work with animals in an agricultural setting [6]. This study did not assess the reasons for, or barriers to, engaging in protective practices, but additional studies on households’ estimation of the risk posed by common exposures to zoonotic pathogens may be able to explain why some protective practices are more widely used than others [50].

### Community-level factors in household participation in protective practices

Knowledge about how zoonotic pathogens enter and spread throughout a community may also influence the types of prevention measures that households take. Primary prevention, aimed at preventing the initial occurrence of disease, requires an understanding of the disease transmission process. In this study, primary prevention activities such as rodent extermination, proper trash disposal, and preventive veterinary care, were less common than secondary prevention measures that are taken in response to a risk like boot-wearing. The specific lack of engagement in preventive veterinary care is of concern because this primary prevention activity reduces the ability of many zoonotic pathogens to initially become established in an animal or human population. Boot and glove wearing around animals and their environment only mitigates transmission between animals and humans, and does not reduce animal infection or any resulting productivity and fertility losses. In addition to a potential knowledge gap surrounding primary zoonotic disease prevention measures, there may be a lack of acceptable or accessible mechanisms through which primary prevention can be conducted. Although the final model for rodent extermination did not identify an association between income and rodent trapping in this analysis, other studies have found that the ability to pay for pest control influences whether a household will engage in protective practices [51, 52]. When gaps in rodent control efforts occur, rodent activity rapidly increases, suggesting that community-wide efforts from the government or external agencies are the best way to ensure continuity in rodent population control [25].

Community-based interventions are similarly recommended for preventive veterinary care, in particular to increase access to veterinary care in low resource rural settings [53–55]. Although in this study income was not associated with participation in preventive veterinary care overall, income may influence more specific outcomes such as preventive veterinary care by type, frequency, or duration. Given the burden that veterinary care can place on livestock owners [9, 15], and that the median monthly income for all households in this study was $350 USD (substantially lower than the national average income of $1146.83 USD per month [56]), it is also possible that there were not enough households with a high enough income to have an impact in veterinary care participation. Further exploration into the types of preventive veterinary care households engaged in, and the access and availability of treatment for livestock and pets, would allow for the identification of conditions in which transmission of zoonotic pathogens may be facilitated.

This study found a great deal of variation between communities and across community types in the proportion of households participating in protective practices. Community type was included as a confounder in the association between the number of rodent signs reported in a household and rodent extermination efforts, as well as the association between number of people in the household and restricting animal access to the garden. Farm communities reported more rodent signs in the household and more people in the household than village and marginalized urban communities (Table 2). Similarly, the farm areas were more likely to participate in rodent extermination efforts (80.9 %) and restricting animal access to the garden (63.6 %) than rural village and marginalized urban communities (Figs. 1 and 3, respectively). Therefore, community type should be considered when studying household-level exposures and outcomes related to zoonotic diseases as the effect of socio-demographic factors are likely affected by the community context in which they exist.

At the community level, the proportion of households within a community that reported participating in rodent extermination efforts ranged from 37.5 % to 90.1 % (Additional file 1). Differences in other rodent control practices were also observed across communities within the same community type. Urban communities had both the highest and lowest proportion of households reporting proper trash disposal, 40.0 % and 6.3 % from U-3 and U-4, respectively (Additional file 1). These two communities were fairly adjacent to one another and shared similar socio-demographic patterns. The micro-scale identity of the communities in this study reinforces the importance of tailoring educational materials and public health outreach efforts to the specific population for which it is intended. There may also be other household and community-level factors not examined in this study that influence household participation in protective practices. Studies have shown that friends and family members, social norms, and media coverage, influence risk perception within a community, and that these factors play a meaningful role in decisions to engage in certain protective behaviors [57–59]. It is also accepted that sociological factors contribute to the transmission of infectious diseases, but difficulty in quantifying the importance of these social networks on individual and household behaviors persists [31].

### Limitations and future research

This study was unable to measure the frequency of duration of the protective practices in which the households participated, which may be influenced by the perceived effectiveness and immediacy in addressing the problems. For example, households with more rodent signs were more likely to report having engaged in rodent extermination efforts. This suggests that the households are trying to resolve a current and immediate rodent infestation issue. It could also be argued that households have rodent signs because they are unsuccessful in their extermination efforts. This temporality dilemma has been reported in other studies of rodent presence and control [60]. Nevertheless, an association between comprehensive rodent control and reduced rodent presence is found throughout the literature, which contributes to lowering human exposure to rodent-borne pathogens [61, 62].

This study also did not consider practices or behaviors that influence the risk of exposure to zoonotic pathogens transmitted from close contact with pets due to limitations in length and depth of the household questionnaire. Pets can be an important source of human infection [63–65]. Awareness of diseases carried by pets is limited among their owners and protective practices such as hand washing after petting or playing with the animals and mindfulness of avoiding areas contaminated with animal feces may not be apparent to pet-owners [63, 66]. Given the role that pets play in contaminating the environment and being accidental hosts for a variety of pathogens [64, 67], identifying gaps in protective practices against zoonotic disease exposure from pets may be just as important as the practices examined in this study for lowering the risk of human infection.

This study analyzed specific protective practices at the household level although it is recognized that protective practices are taken by individuals. It was assumed that risk perception and practices were highly correlated among household members who engaged in the same activities, and household-level data have been used successfully to examine factors associated with zoonotic disease risk in other communities[68, 69]. To better capture the individual dynamics that contribute to household engagement in protective practices, additional investigations can be conducted using mixed methods approaches. Qualitative data would be particularly useful in evaluating the knowledge and perceptions on zoonotic disease susceptibility and barriers to engaging in protective practices.

## Conclusions

This study found that participation in recommended protective practices against zoonotic disease transmission including rodent control, occupational protection, and protection in the garden, is sub-optimal among the study population and varied substantially by community type and across the twelve communities included in the study. Results suggest that, in this population, inter-community differences influence household participation in protective practices, may be more than household socio-demographic characteristics. Consequently, education campaigns could be tailored to specific communities in an effort to expand the use of recommended practices. Further research on social and culture determinants such as shared norms, beliefs, and knowledge about zoonotic disease transmission is also needed to better understand the reasons why households do or do not participate in specific practices. The survey data used in this study would be well-complemented by qualitative research that addresses the duration and frequency of household participation in protective practices against exposure to zoonotic pathogens, and the barriers to and perceived benefits of engaging in such practices.

## Additional file

Additional file 1:
**Participation in protective practices against zoonotic disease transmission by community, Los Rios Region, Chile (2010–2012).** The table displays the proportion of households in each of the 12 communities (rural villages: C1–C4, farm communities: D1–D4, and marginalized urban communities: U1–U4) that reported participation in protective practices in the three categories of rodent control, occupational protection, and garden protection. All households were included in the assessment of rodent control, farm households were included in the assessment of occupational protection, and households with gardens and livestock were included in the assessment of garden protective practices.
