# Supplementary material for: Protective practices against zoonotic infections among rural and slum communities from South Central Chile
Source: BMC Public Health. 2015 Jul 28;15:713. doi: 10.1186/s12889-015-1964-2 (PMC4517625; doi:10.1186/s12889-015-1964-2)
Supplement: Additional file 1: — Participation in protective practices against zoonotic disease transmission by community, Los Rios Region, Chile (2010–2012). The table displays the proportion of households in each of the 12 communities (rural villages: C1–C4, farm communities: D1–D4, and marginalized urban communities: U1–U4) that reported participation in protective practices in the three categories of rodent control, occupational protection, and garden protection. All households were included in the assessment of rodent control, farm households were included in the assessment of occupational protection, and households with gardens and livestock were included in the assessment of garden protective practices. [file 12889_2015_1964_MOESM1_ESM.docx]

| **Reported Protective Practices** | **Community** | | | | | | | | | | | | **Community type P-Value** | |
| --- | --- | --- | --- | --- | --- | --- | --- | --- | --- | --- | --- | --- | --- | --- |
|  | C-1 | C-2 | C-3 | C-4 | D-1 | D-2 | D-3 | D-4 | U-1 | U-2 | U-3 | U-4 | D v. C | U v. C |
| **Rodent Control**  **(n=422 Households)** | (n=30) | (n=31) | (n=38) | (n=35) | (n=41) | (n=30) | (n=40) | (n=35) | (n=40) | (n=40) | (n=30) | (n=32) |  |  |
| % Rodent Extermination | 73.3 | 67.7 | 55.3 | 51.4 | 73.1 | 90.1 | 77.5 | 85.7 | 57.5 | 65.0 | 63.3 | 37.5 | 0.01 | 0.44 |
| % Food in Closed Container | 96.7 | 90.3 | 86.8 | 94.3 | 90.2 | 93.3 | 95.0 | 85.7 | 72.5 | 92.5 | 90.0 | 90.6 | 0.84 | 0.18 |
| % Proper Trash Disposal | 26.7 | 12.9 | 10.5 | 22.9 | 12.2 | 13.3 | 10.0 | 14.3 | 17.5 | 12.5 | 40.0 | 6.3 | 0.28 | 0.95 |
| **Occupation Protection**  **(n=110 Households)** |  |  |  |  | (n=37) | (n=26) | (n=36) | (n=28) |  |  |  |  |  |  |
| % Preventive Veterinary Care |  |  |  |  | 21.6 | 38.5 | 19.4 | 0.0 |  |  |  |  | N/A | N/A |
| % Boot Wearing |  |  |  |  | 78.4 | 73.1 | 47.2 | 3.6 |  |  |  |  | N/A | N/A |
| % Glove Wearing |  |  |  |  | 35.1 | 46.2 | 8.3 | 7.1 |  |  |  |  | N/A | N/A |
| **Garden Protection**  **(n=233 Households)** | (n=21) | (n=22) | (n=29) | (n=15) | (n=34) | (n=28) | (n=35) | (n=24) | (n=2) | (n=5) | (n=5) | (n=13) |  |  |
| % Animal Access Prohibited | 47.6 | 31.8 | 48.3 | 66.7 | 73.5 | 71.4 | 48.6 | 62.5 | 50.0 | 20.0 | 40.0 | 7.7 | 0.03 | 0.03 |
| % Boot Wearing | 76.2 | 31.8 | 20.7 | 20.0 | 91.2 | 67.9 | 40.0 | 37.5 | 50.0 | 0.0 | 80.0 | 7.7 | 0.50 | 0.72 |
| % Glove Wearing | 28.6 | 18.2 | 27.6 | 20.0 | 32.4 | 28.6 | 14.3 | 0.0 | 0.0 | 0.0 | 80.0 | 7.7 | 0.13 | 0.60 |

The P-values presented for community type come from the regression model for the protective practice of interest with the independent variable of community type. The regression model includes random intercepts for individual communities. Community type was an important covariate in the model for rodent extermination and the model for prohibiting animal access to the garden. In most other models the variation in the proportion of households in each community participating in a protective practice was larger than the variation across community types.
